# Microsatellite Status Affects Tumor Response and Survival in Patients Undergoing Neoadjuvant Chemotherapy for Clinical Stage III Gastric Cancer

**DOI:** 10.3389/fonc.2020.614785

**Published:** 2020-12-15

**Authors:** Zhenghao Cai, Weiwei Rui, Shuchun Li, Abraham Fingerhut, Jing Sun, Junjun Ma, Lu Zang, Zhenggang Zhu, Minhua Zheng

**Affiliations:** ^1^ Department of General Surgery, Ruijin Hospital, Shanghai Jiao Tong University School of Medicine, Shanghai, China; ^2^ Shanghai Minimally Invasive Surgery Center, Shanghai, China; ^3^ Department of pathology, Ruijin Hospital, Shanghai Jiao Tong University School of Medicine, Shanghai, China; ^4^ Section for Surgical Research, Department of Surgery, Medical University of Graz, Graz, Austria

**Keywords:** gastric cancer, microsatellite instability, neoadjuvant chemotherapy, tumor response, curative gastrectomy

## Abstract

**Background:**

We assessed the association between microsatellite instability-high (MSI-H) and tumor response to neoadjuvant chemotherapy (NAC) as well as its prognostic relevance in patients with clinical stage III gastric cancer (cStage III GC).

**Materials and Methods:**

The NAC + surgery and the control cohorts consisted of 177 and 513 cStage III GC patients, respectively. The clinical and pathological features were compared between patients with MSI-H [n=57 (8.3%)] and microsatellite stability or microsatellite instability-low (MSS/MSI-L) [n=633 (91.7%)]. Radiological and histological response to NAC were evaluated based on response evaluation criteria in solid tumors (RECIST) and tumor regression grade (TRG) systems, respectively. The log-rank test and Cox analysis were used to determine the survival associated with MSI status as well as tumor regression between the two groups in both NAC + surgery and the control cohorts.

**Results:**

A statistically significant association was found between MSI-H and poor histological response to NAC (*p*=0.038). Significant survival priority of responders over poor-responders could only be observed in MSS/MSI-L but not in MSI-H tumors. However, patients with MSI-H had statistically significantly better survival compared to patients with MSS/MSI-L in both the NAC + surgery (hazard ratio=0.125, 95% CI, 0.017–0.897, *p*=0.037 ) and the control cohort (hazard ratio=0.479, 95% CI, 0.268–0.856, *p*=0.013).

**Conclusion:**

MSI-H was associated with poorer regression and better survival after NAC for cStage III GC. TRG evaluation had prognostic significance in MSS/MSI-L but not in MSI-H. Further studies are needed to assess the value of NAC for cStage III GC patients with MSI-H phenotype.

## Introduction

Gastric cancer (GC) is the third most common cause of cancer-related death worldwide (1). While radical gastrectomy with lymphadenectomy remains the cornerstone of curative treatment of GC, prognosis is still poor with a high recurrence rate, especially for patients with locally advanced and/or lymph node (LN) positive cancer, leading to recommendations for routine perioperative or neoadjuvant chemotherapy (NAC) for resectable stage II or III GCs (2–4). However, the selection of patients who might most benefit from NAC is based purely on radiologic staging. Few biomarkers of GC have been identified to predict the response to NAC.

There is increasing interest in the potential value of molecular subtypes and particularly, microsatellite instability-high (MSI-H) as a prognostic marker (5–7). Conflicting results have been reported with regard to the prognostic significance of MSI status for GC patients treated with NAC (8–11). The review by Ratti et al. suggested that outcome was worse after perioperative chemotherapy, related to a detrimental role of cytotoxic drugs in MSI-H subgroup (12). However, the heterogeneity of clinical stages (II and/or III) included in the studies makes it difficult to interpret the data. Moreover, few studies have assessed the impact of MSI phenotype on tumor response to NAC (*i.e.* histopathological regression of GC after NAC) (13).

According to the Chinese Society of Clinical Oncology (CSCO) guideline, NAC is recommended for cT_3-4a_N_+_M_0_ GC or cT_4a_N_any_M_0_ adenocarcinoma of the esophago-gastric junction (AEG) (*i.e.* clinical stage III patients) (14). Our aim was to investigate the association between MSI-H and tumor response to NAC as well as its prognostic relevance in patients with clinical stage (cStage) III GC treated by NAC and curative gastrectomy.

## Material and Methods

Clinical and pathological data were retrospectively collected from our prospective institutional database. All procedures followed were in accordance with the ethical standards of our institutional ethics committee and with the Helsinki Declaration of 1964.

### Patients

All patients treated by NAC and then curative gastrectomy for cStage III GC or AEG (adenocarcinoma, mucinous adenocarcinoma, and signet ring cell carcinoma) at Ruijin Hospital between February 2016 and June 2018 were eligible for this study. The control group consisted of patients treated by curative surgery ± adjuvant chemotherapy but without NAC, for cStage III GCs or AEGs during the same period in our institution. Patients with squamous cell carcinoma, lymphoma, gastrointestinal stromal tumor, neuroendocrine tumor, GC related to other malignancies, AEG type I [according to Siewert et al. (15)], those treated with palliative resection or emergency procedures, or patients with incomplete postoperative pathological evaluation records were not included.

### Initial Diagnosis and Neoadjuvant Chemotherapy Protocol 

Esophagus-gastro-duodenoscopy with biopsy and thoracic-abdominal-pelvic computed tomography (TAP-CT) were routinely performed to obtain histological diagnosis and determine tumor staging, respectively. In line with the CSCO guideline (14), three to four cycles of platinum/5-fluorouracil based doublet or triplet NAC regimens were administered to patients with cStage III GC/AEG according to the Union for International Cancer Control (UICC)/American Joint Committee on Cancer (AJCC) TNM classification, 8^th^ edition (16). A regimen containing Apatinib was administered to 22 patients in this cohort, who were participants of a prospective, single arm, phase II trial conducted in our institution at the same period (17). Tumor response after NAC was assessed on control TAP-CT by a dedicated radiologist, who was unaware of the future inclusion of patients in this study, using the Response Evaluation Criteria in Solid Tumors (RECIST) guideline version 1.1 (18), and recorded as complete response (CR: disappearance of all target lesions), partial response (PR: at least a 30% decrease in the sum of diameters of target lesions), progressive disease (PD: at least a 20% increase in the sum of diameters of target lesions or the appearance of new lesions) or stable disease (SD: between PR and PD). The objective response rate (ORR) was defined as the proportion of patients who had CR or PR (18).

### Surgical Procedure

All patients underwent laparoscopic exploration to confirm tumor resectability; curative gastrectomy was performed either by laparoscopy or after conversion to open surgery. Distal/total gastrectomy (DG/TG) + D2 lymphadenectomy was routinely performed for GC. For AEG types II and III, a transhiatal extended gastrectomy (THG) with mediastinal lymphadenectomy was performed.

#### Pathological Evaluation and Tumor Regression

Tumors were staged post-operatively according to the UICC/AJCC (16). Histological categorization was documented according to Lauren’s classification (19). Tumor response to NAC was evaluated based on tumor regression grade (TRG) described by Becker et al. : Grade 1, complete or subtotal regression (<10% residual tumor per tumor bed; Grade 1a, complete regression and Grade 1b, subtotal regression); Grade 2, partial tumor regression (10%–50% residual tumor per tumor bed); and Grade 3, minimal or no tumor regression (>50% residual tumor per tumor bed) (20). In accordance with Becker et al. (21), Grade 1 patients were defined as responders and those with Grades 2 or 3 as poor-responders.

### Microsatellite Status Analysis

The MSI status of the surgical specimens was determined using a five-Bethesda-marker (NR-24, BAT-25, BAT-26, CAT-25, MONO-27) panel (22). Tumors with instability at two or more of the five markers were classified as MSI-H. Those with one unstable marker were classified as microsatellite instability-low (MSI-L) whereas tumors with all five markers stable were classified as microsatellite stability (MSS).

### Expression of Mismatch Repair Proteins

Four MMR proteins, MLH1, MSH2, PMS2, and MSH6, were submitted to immunohistochemical staining on formalin-fixed, paraffin-embedded tumor tissue sections. The loss of expression of a single protein or a dimeric couple (MLH1/PMS2 or MSH2/MSH6) suggested the presence of MMR deficiency (dMMR) (12).

### Adjuvant Therapy and Follow-Up

Patients were given postoperative chemotherapy based on age, pathological results as well as their Eastern Cooperative Oncology Group (ECOG) performance. All patients were followed every month during the first year, then every 3 months during the second and the third years, then every 6 months until recurrence or the censoring date. A multidisciplinary team managed patients with recurrence and/or metastasis. Serum tumor markers (CEA, CA199, CA125) were obtained every 3 months during the first 3 years then at every visit. TAP-CT and endoscopy were conducted every 6 months during the first 2 years then annually after surgery. Disease-free survival (DFS) was defined as the time between the date of surgery and the date either of recurrence or death from any cause.

### Statistical Analysis

Statistical analysis was performed using the IBM Statistical Package for the Social Sciences (SPSS 13.0, Chicago, IL, USA). Pearson’s Chi-Square or Fisher’s exact test was applied for categorical data, expressed as percentages. For median and quartiles, the non-parametric Wilcoxon rank-sum test was used. The median DFS was determined by the Kaplan-Meier method, and survival curves were compared with the log-rank test. Cox analysis was used to determine the survival associated with MSI status as well as tumor regression. Two-sided *p*-values <0.05 were considered to be statistically significant.

## Results

### Patient Enrollment

**Figure 1:**
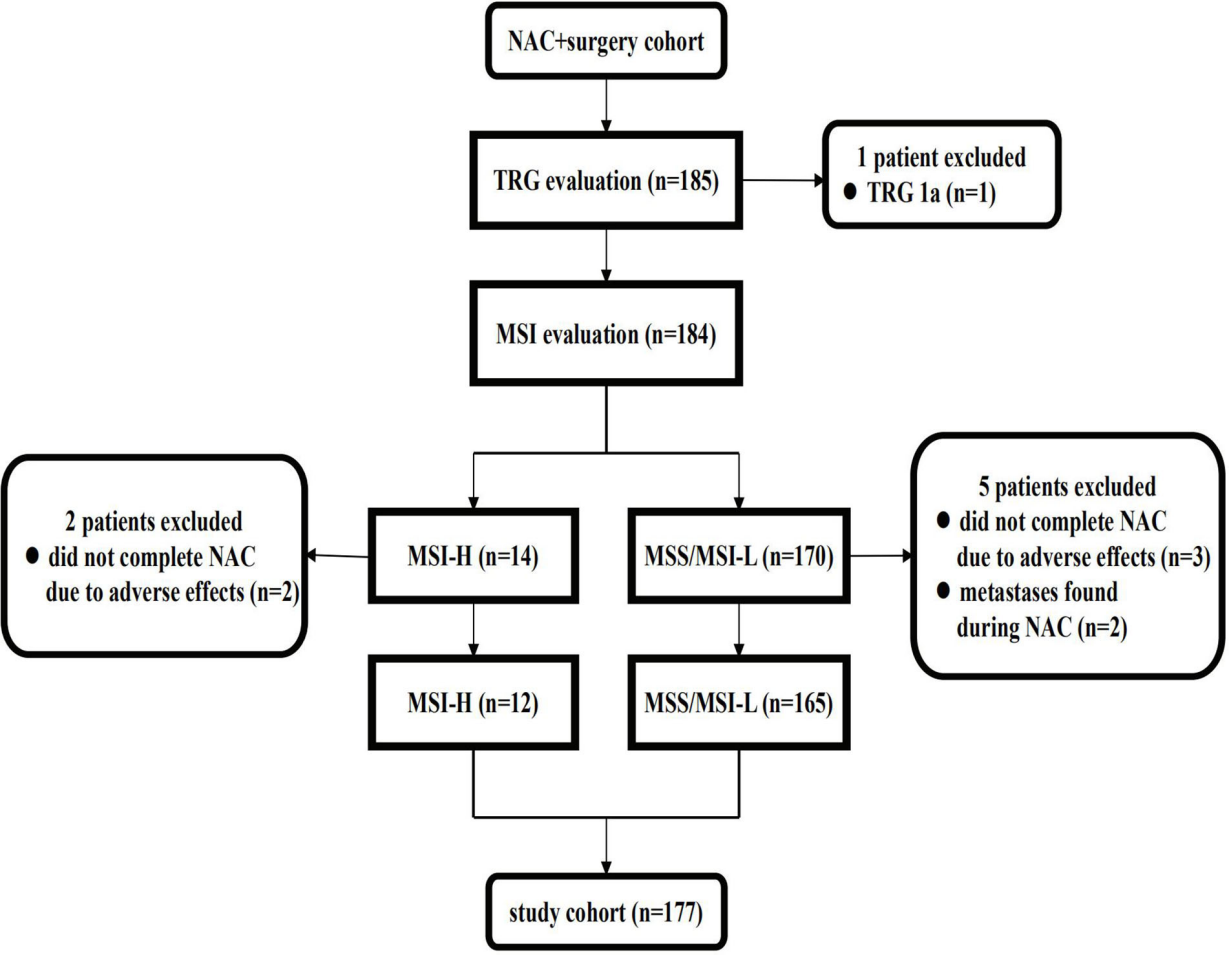
Flow diagram of patients excluded from the NAC cohort. NAC, neoadjuvant chemotherapy; TRG, tumor regression grade; MSS, microsatellite stability; MSI-L, microsatellite instability-low; MSI-H, microsatellite instability-high.

The NAC + surgery cohort included 130 (70.3%) males and 55 (29.7%) females [median age: 61 (range, 22–81) years]. Microsatellite status and MMR analysis were available for 184/185 specimens (no residual tumor tissue available (TRG 1a) in one patient). As seen in the flow diagram (**Figure 1**), eight patients were excluded from this cohort. Thus, 177 patients were retained for survival and tumor regression analysis. Details of NAC regimens administered are shown in **Table 1**. Postoperative chemotherapy regimens were: EOX (107/177), CapeOX/SOX (55/177), DS/PS (5/177) or Capecitabine/S-1 (10/177). The same chemotherapy regimen was given pre- and post-operatively to 124/177 patients. An adjuvant regimen different from NAC was given to patient either because he presented PD during NAC or because he was given a pre-operative SOXA + post-operative SOX regimen according to the protocol of our Apatinib phase II trial (17).

**Table 1:** Neoadjuvant chemotherapy regimens.

Regimens, n (%)	MSI-H	MSS/MSI-L
	*n* = 12 (6.8%)	*n* = 165 (93.2%)
Oxaliplatin + Capecitabine/S-1(CapeOX/SOX)	1 (8.3)	14 (8.5)
Docetaxel + oxaliplatin + 5-FU/S-1 (FLOT/DOS)	0 (0)	21 (12.7)
Epirubicin + oxaliplatin + capecitabine (EOX)	8 (66.7)	99 (60.0)
Apatinib + oxaliplatin + S-1 (SOXA)	1 (8.3)	21 (12.7)
Docetaxel/Paclitaxel + S-1 (DS/PS)	1 (8.3)	6 (3.6)
Other platinum/5-FU based regimens	1 (8.3)	4 (2.4)

MSI-H, microsatellite instability-high; MSS, microsatellite stability; MSI-L, microsatellite instability-low.

The cohort without NAC included 354 (69.0%) males and 159 (31.0%) females [median age: 64 (range, 26–90) years]. Microsatellite status and MMR analysis were available for all 513 specimens in this control cohort.

As of June. 2020, eight patients (one in the NAC + surgery cohort, seven in the control cohort) were lost to follow-up. The median follow-up of the whole cohort (n=690) was 27.3 (1.5–51.9) months.

### Microsatellite Status and Expression of Mismatch Repair Proteins

MSI-H status was identified in 57 (8.3%) patients and dMMR was found in all these MSI-H tumors (loss of expression of the MLH1/PMS2 dimeric couple in 55 patients and loss of expression of PMS2 protein in two patients). However, seven patients with the same patterns of dMMR and five patients with other patterns were found to be MSS/MSI-L by the five-Bethesda-marker panel. There was no significant difference in the prevalence of MSI-H, dMMR or the loss of expression of four MMR proteins between the NAC + surgery cohort and the control cohort.

### Patient Demographics and Pathological Evaluation

Clinical characteristics of 57 MSI-H and 633 MSS/MSI-L patients are summarized in **Table 2**. MSI-H was more frequently observed among females and elderly patients. No statistically significant difference was found between the two groups with regard to tumor location, cT stage and cN stage. The ORR after NAC was 55.9% (99/177): 33.3% (4/12) for MSI-H and 57.6% (95/165) for MSS/MSI-L, showing no statistically significant difference (*p*=0.102). In terms of pathological evaluation (**Table 3**), a statistically significant difference was found between the two groups with regard to tumor size, Lauren’s classification, perineural invasion, pN stage (for patients in the control cohort) and AJCC/UICC p stage (for patients in the control cohort). In the NAC + surgery cohort, all MSI-H patients were poor-responders to NAC, whereas 46 (27.9%) and 119 (72.1%) patients in the MSS/MSI-L group were considered as responders and poor-responders, respectively (*p*=0.038). Overall, responders in TRG evaluation was positively correlated to radiological tumor response (CR + PR) (r=0.267, p<0.001, Pearson correlation analysis).

**Table 2:** Patient demographics, clinical patterns, and microsatellite status.

	MSI-H	MSS/MSI-L	*p* value
	*n* = 57 (8.3%)	*n* = 633 (91.7%)	
Sex, n (%)			*0.010*
Male	31 (54.4)	448 (70.8)	
Female	26 (45.6)	185 (29.2)	
Age [y], median (quartile)	68 (61-75)	63 (56–69)	*0.003*
Tumor location, n (%)			0.083
GC	53 (93.0)	531(83.9)	
AEG	4 (7.0)	102 (16.1)	
Extent of resection, n (%)			*<0.001*
DG	39 (68.4)	212 (33.5)	
TG/THG	18 (31.6)	421 (66.5)	
Surgical procedure, n (%)			0.645
Open surgery	43 (75.4)	453 (71.6)	
Laparoscopic surgery	14 (24.6)	180 (28.4)	
cT stage, n (%)			0.337
T_≤2_	0 (0.0)	2 (0.3)	
T_3_	8 (14.0)	139 (22.0)	
T_4a_	49 (86.0)	492 (77.7)	
cN stage, n (%)			0.502
N_0_	1 (1.8)	29 (4.6)	
N_+_	56 (98.2)	604 (95.4)	
Adjuvant chemotherapy			0.598
Yes	42 (73.7)	486 (76.8)	
No/not completed	15 (26.3)	147 (23.2)	
^†^ Response Evaluation, n (%)	*n* = 12 (6.8%)	*n* = 165 (93.2%)	0.102
CR/PR (ORR)	4 (33.3)	95 (57.6)	
SD/PD	8 (66.7)	70 (42.4)	

† only included patients in the NAC + surgery cohort.

MSI-H, microsatellite instability-high; MSS, microsatellite stability; MSI-L, microsatellite instability-low; GC, gastric cancer; AEG, adenocarcinoma of esophago-gastric junction; DG, distal gastrectomy; TG, total gastrectomy; THG, transhiatal extended gastrectomy; ORR, objective response rate; CR, complete response; PR, partial response; SD, stable disease; PD, progressive disease; NAC, neoadjuvant chemotherapy.

**Table 3:** Pathological characteristics and microsatellite status.

	MSI-H	MSS/MSI-L	*p* value
	*n* = 57 (8.3%)	*n* = 633 (91.7%)	
Tumor size [cm], median (quartile)	6 (4.5-7.5)	4.5 (3-6)	*<0.001*
Histological type, n (%)			0.548
Well differentiated	16 (28.1)	155 (24.5)	
Moderate/poor differentiated	41 (71.9)	478 (75.5)	
Lauren’s classification, n (%)		(Missing = 1)	*<0.001*
intestinal type	41 (71.9)	220 (34.8)	
diffuse type	7 (12.3)	324 (51.3)	
mixed type	9 (15.8)	88 (13.9)	
Lymphovascular emboli, n (%)			0.250
No	31 (54.4)	294 (46.4)	
Yes	26 (45.6)	339 (53.6)	
Perineural invasion, n (%)			*<0.001*
No	39 (68.4)	241 (38.1)	
Yes	18 (31.6)	392 (61.9)	
Total lymph node count, median (quartile)	32 (26-39)	31 (23-41)	0.824
^#^ pT stage, n (%)	*n* = 45 (8.8%)	*n* = 468 (91.2%)	1.000
T_≤2_	0 (0.0)	6 (1.3)	
T_≥3_	45 (100.0)	462 (98.7)	
^#^ pN stage, n (%)	*n* = 45 (8.8%)	*n* = 468 (91.2%)	*<0.001*
N_0_	9 (20.0)	20 (4.3)	
N_+_	36 (80.0)	448 (95.7)	
^#^ AJCC/UICC pStage, n (%)	*n* = 45 (8.8%)	*n* = 468 (91.2%)	*<0.001*
II	14 (31.1)	32 (6.8)	
III	31 (68.9)	436 (93.2)	
^†^ ypT stage, n (%)	*n* = 12 (6.8%)	*n* = 165 (93.2%)	0.311
T_≤2_	5 (41.7)	43 (26.1)	
T_≥3_	7 (58.3)	122 (73.9)	
^†^ ypN stage, n (%)	*n* = 12 (6.8%)	*n* = 165 (93.2%)	0.095
N_0_	6 (50.0)	43 (26.1)	
N_+_	6 (50.0)	122 (73.9)	
^†^ AJCC/UICC ypStage, n (%)	*n* = 12 (6.8%)	*n* = 165 (93.2%)	0.267
I	4 (33.3)	26 (15.6)	
II	3 (25.0)	41 (24.6)	
III	5 (41.7)	98 (58.7)	
^†^ TRG, n (%)	*n* = 12 (6.8%)	*n* = 165 (93.2%)	*0.038*
Responders (Grade 1b)	0 (0.0)	46 (27.9)	
Poor-responders (Grades 2&3)	12 (100.0)	119 (72.1)	

# only included patients in the control cohort.

† only included patients in the NAC + surgery cohort.

MSI-H, microsatellite instability-high; MSS, microsatellite stability; MSI-L, microsatellite instability-low; TRG, tumor regression grade; NAC, neoadjuvant chemotherapy.

### Prognostic Value of Microsatellite Instability-High and MMR Deficiency

**Figure 2:**
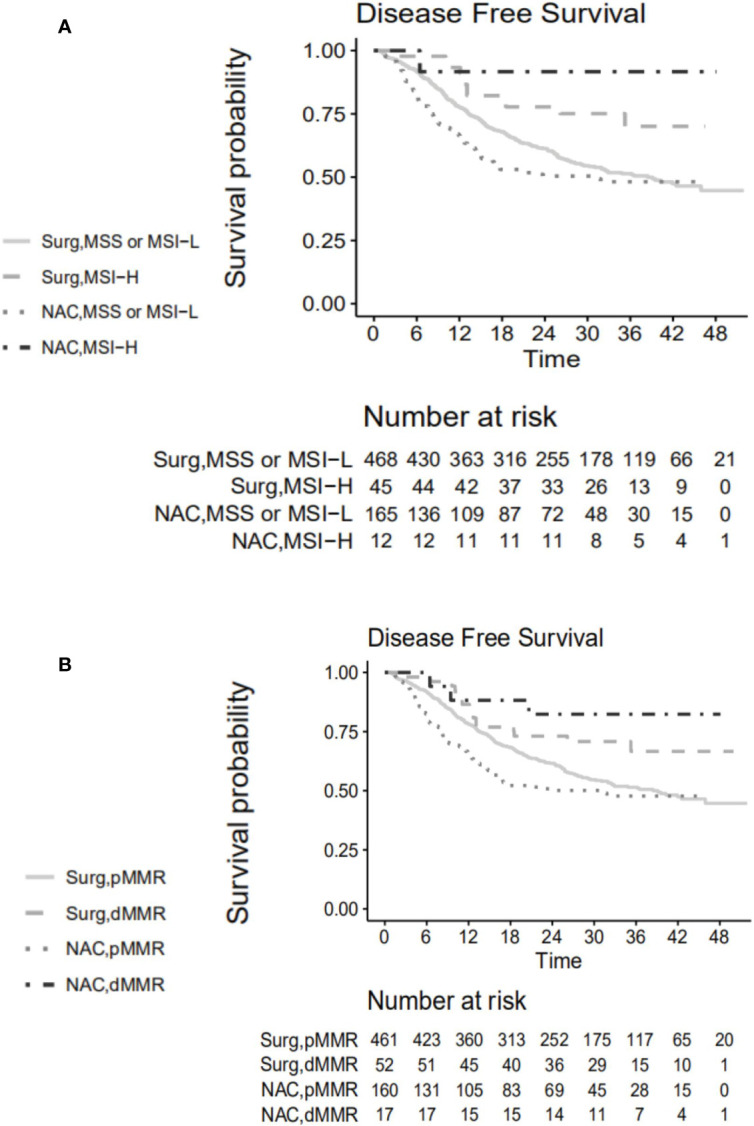
Disease-free survival curve according to **(A)** microsatellite status. **(B)** expression of mismatch repair proteins. Surg, the control group; NAC, the neoadjuvant chemotherapy + surgery group; MSS, microsatellite stability; MSI-L, microsatellite instability-low; MSI-H, microsatellite instability-high; pMMR, mismatch repair proficiency; dMMR, mismatch repair deficiency.

The 3-year DFS rates differed statistically significantly between MSI-H and MSS/MSI-L patients in both the NAC + surgery cohort (91.7% *vs.* 48.2%, respectively) and the control cohort (70.1% *vs.*51.4%, respectively) (**Figure 2A**). The hazard ratios for MSI-H *vs.* MSS/MSI-L were 0.125 (95% CI, 0.017-0.897) for the NAC + surgery (*p*=0.037) and 0.479 (95% CI, 0.268–0.856) for the control cohort (*p*=0.013). The 3-year DFS rates differed statistically significantly between dMMR and pMMR (MMR proficiency, expression of all four MMR proteins) patients in both the NAC + surgery cohort (82.4% *vs.* 47.8%, respectively) or the control cohort (66.6% *vs.*51.5%, respectively) (**Figure 2B**). The hazard ratios for dMMR *vs.* pMMR were 0.264 (95% CI, 0.083–0.837) for the NAC + surgery (*p*=0.023) and 0.576 (95% CI, 0.347–0.957) for the control cohort (*p*=0.033).

### Prognostic Value of Tumor Regression Grade

**Figure 3:**
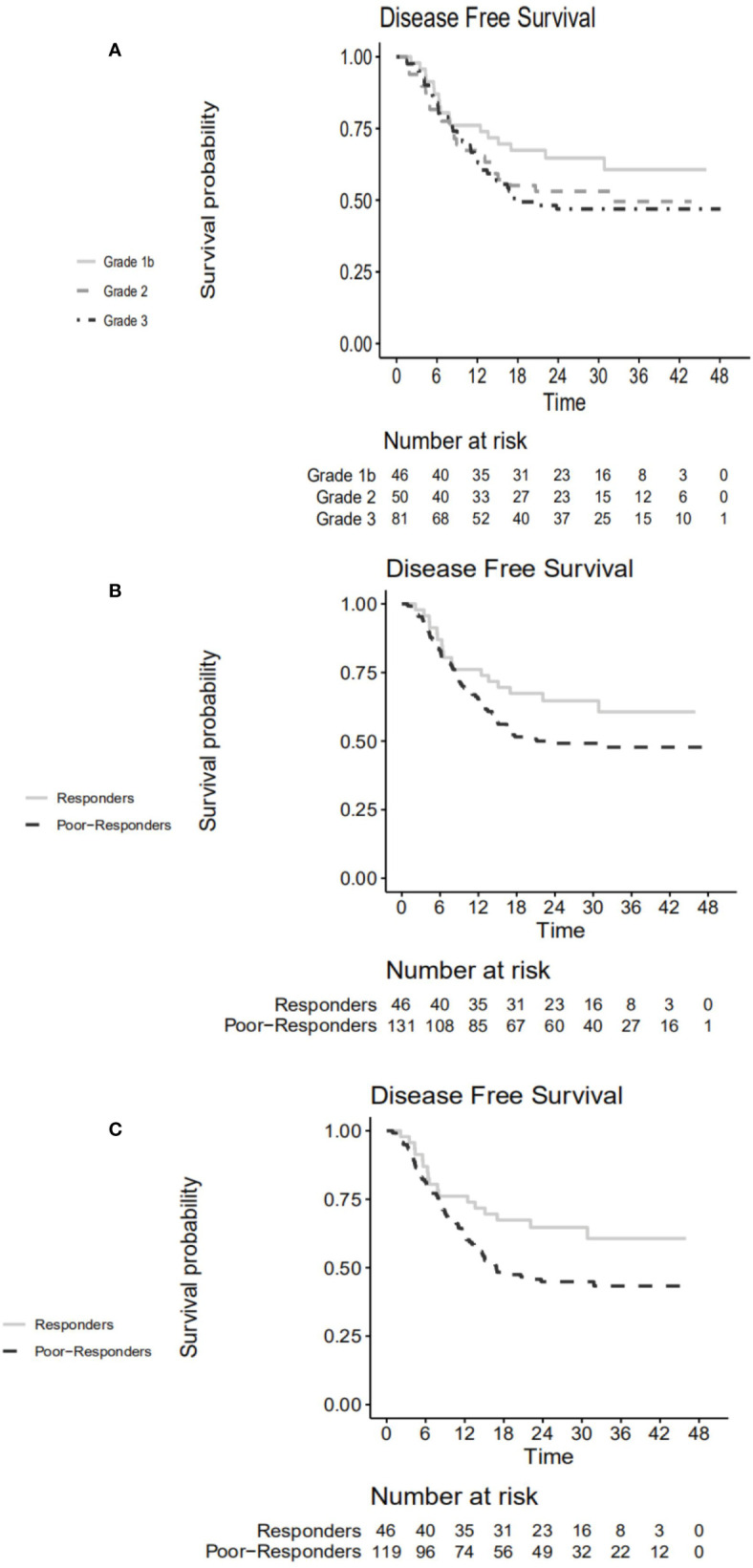
Disease-free survival curve according to **(A)** TRG in the NAC + surgery cohort. **(B)** responders *vs.* poor-responders in the NAC + surgery cohort. **(C)** responders *vs.* poor-responders in MSS/MSI-L group. TRG, tumor regression grade; NAC, neoadjuvant chemotherapy; MSS, microsatellite stability; MSI-L, microsatellite instability-low.

Survival curves stratified according to TRG are shown in **Figure 3A**. Responders tended to have higher 3-year DFS rate compared to poor-responders (60.7% *vs.* 47.8%, *p*=0.110) (**Figure 3B**). The hazard ratio for responders *vs.* poor-responders was 0.651 (95% CI, 0.379–1.110, *p*=0.115) in the NAC + surgery cohort. However, the 3-year DFS rates differed statistically significantly between responders and poor-responders in patients who were MSS/MSI-L (60.7% *vs.* 43.3%, *p*=0.042) (**Figure 3C**). The hazard ratio for responders *vs.* poor-responders was 0.579 (95% CI, 0.340–0.907, *p*=0.045) in the MSS/MSI-L group.

## Discussion

Our study found a statistically significant association between microsatellite instability and poor histological response to NAC in cStage III GC. Significant survival priority of responders to NAC could be observed in MSS/MSI-L group but not in MSI-H group where all patients were poor-responders. We hypothesize that TRG would probably be inapplicable to predict prognosis for this molecular subtype of GC. On the other hand, MSI-H was found to be a positive survival predictor in cStage III GC patients irrespective of whether NAC was given or not. Compared to the control cohort, the better survival of MSI-H patients in the NAC + surgery cohort suggests that cStage III GC patients with this molecular alteration might still benefit from NAC in spite of poor histological response.

The prevalence of MSI-H in our study (8.3%) was lower than the 9.2% overall rate reported in the meta-analysis by Polom et al. (23). As MSI-H is associated with earlier stage at diagnosis and limited nodal metastasis (12), this might reasonably explain the difference since only cStage III patients were included in our study. In previous studies, the prevalence of MSI-H in GC patients treated by NAC varied from 6.6% to 9.0% (8, 10, 11). The lack of standardized diagnostic criteria (different marker panels used to detect MSI status) might be responsible for this heterogeneity. The absence of MLH-1 expression was observed in 62 out of 690 (9.1%) patients, close to the 9.8% (28/285) rate reported by Hashimoto et al. (9), but higher than those in two European studies [5.2% (8) and 7.9% (11), respectively]. Differences in ethnicity (Asian *vs.* Caucasian) are a probable explanation. The close prevalences of MSI-H/dMMR in the NAC + surgery and the control cohorts in our study seemed to indicate that NAC probably does not influence microsatellite status or the expression of MMR proteins.

Several well-recognized associations between clinical/pathological features and MSI-H (female sex, older age, occurrence in stomach rather than EG junction, larger tumor size, perineural invasion (+), intestinal type) were found in our study (8, 11, 24). MSI-H tumors were located more frequently in the distal stomach (24), which explained why the proportion of distal gastrectomy was higher in MSI-H group compared to MSS/MSI-L group. Kohlruss et al. found that MSI-L was more frequent in intestinal GC (10). This discrepancy suggests that the relationship between microsatellite status and Lauren’s classification as well as its prognostic significance need to be further investigated for locally advanced GCs. After NAC, the ORR of the whole cohort was 55.9% (99/177), higher than 37.3% (25/67) reported by Achilli et al. (25). However, the NAC regimens were different and these authors included cStage II tumors (51%) whereas we did not. In our study, tumor response assessed by Recist 1.1 criteria showed a good correlation to pathological evaluation of tumor response to NAC (TRG system), which was in line with a study conducted in rectal cancer (26). Whether MSI-H status would influence the radiologic response evaluated by RECIST criteria was unclear since we were unable to find any relevant studies to compare with ours. In accordance with other studies (8, 11), no statistically significant differences were found in pathological stages (ypT, ypN) after NAC between the two groups. However, for patients in the control cohort, the proportions of pN_0_ stage and AJCC/UICC pStage II were higher in MSI-H group compared to MSS/MSI-L group (20.0% *vs.* 4.3%, 31.1% *vs.* 6.8%, both *p*<0.001). This statistically significant difference suggests that the MSI-H tumors are prone to up-staging (especially N_0_ to N_+_) by preoperative TAP-CT. A reasonable explanation could be that the average size of lymph nodes in MSI-H was much larger than that in MSS/MSI-L cancers (27).

MSI-H’s poor histological response to NAC has been observed previously but the difference in response rates was not statistically significant (*p*=0.36 and *p*=0.683, respectively) (8, 11). Hashimoto et al. considered the loss of MLH-1 expression as a predictor of poor histological regression after NAC evaluated by the Japanese Gastric Cancer Association criteria (9). However, they did not investigate the association between MSI-H and tumor regression. Kohlruss et al. observed a higher proportion of TRG 3 (*p*=0.002) in MSI-H patients but found that MSI-H was not associated with poor response to NAC (*p*=1.00) (10). To the best of our knowledge, our study is the first to report a statistically significant association between poor histological response to NAC (TRG 2 or 3) and MSI-H.

Despite the poor histological response to NAC, the benefit of MSI-H did not seem to be attenuated by peri-operative chemotherapy in cStage III gastric cancer. In our study, patients with MSI-H had a statistically significant better 3-year DFS rate compared to MSS/MSI-L after NAC. This contradictory association is in line with two (10, 11), but in opposition to two other studies (8, 9), one of which was a post-hoc study of the MAGIC trial (2). There are several hypotheses that might possibly explain these divergent results. First, tumor stage varied in these studies. More ypT_≤2_ patients were included in the MAGIC trial (37.7% in the whole cohort and 55.5% in MSI-H group) whereas the proportions were 26.3% in our study, and 23.8%, 24.2%, 14.1% in other studies, respectively (9, 10, 11). Regardless of the down-staging effect of different regimens applied in these studies, we can speculate that indications for NAC in the MAGIC trial were more liberal compared to the other studies. The negative outcome could be explained by the higher proportion of less-advanced GCs. On the other hand, the Japanese study included patients with metastases (13.6% in MSS/MSI-L and 7.1% in MSI-H groups, respectively), although the authors claimed that these patients underwent R_0_ resection after NAC (9). This relatively high proportion of stage IV patients could affect the survival analysis, overshadowing the potential survival priority of patients with MSI-H. In our study, only clinical stage III patients were included, resulting in a homogeneous tumor stage. Secondly, different NAC regimens were administered. A platinum/5-fluorouracil based regimen ± anthracycline was used in the two studies with negative results (8, 9), while in the other studies (10, 11) and ours, some patients received a taxane (Docetaxel/Paclitaxel)-based regimen. Indeed, several studies have reported resistance of MSI-H tumors to fluorouracil-based chemotherapy and platinum drugs (28, 29). A post-hoc study of the CLASSIC trial also demonstrated that a capecitabine plus oxaliplatin-based adjuvant chemotherapy was of no benefit for MSI-H patients with stage II to III disease (30). Tsai et al. owed this MSI-relevant chemoresistance to increased autophagy activation (31). However, Nikanjam et al. found that MSI-H was correlated to the low expression of TUBB3, a protein biomarker associated with taxane resistance (32). Although stratified analysis according to regimens was unavailable in our study due to the small sample size in MSI-H group, we can only speculate that taxane-based regimens might improve survival of MSI-H patients. Thirdly, the disparity of diagnostic criteria for MSI status reduced the comparability of populations since no two studies applied identical panels to detect MSI-H. Of note, the Japanese study used an IHC method to test the loss of MLH1 expression; however, 14.3% of the MLH1 negative tumors were found to be MSS/MSI-L in their study (9).

In our study, MSI-H patients receiving NAC + surgery showed a better (although not statistically significant) survival compared to MSI-H patients with surgery alone. Whether or not MSI-H patients benefit from NAC has widely been discussed in GC. According to results of a large-sample-size individual patient data meta-analysis, Pietrantonio et al. suggested the possibility of omission of chemotherapy in MSI-H GCs according to a clinically and pathologically defined risk of relapse (33). Currently, MSI-H status is routinely taken into consideration in deciding whether chemotherapy should be administered or not in stage II (but not stage III) colorectal cancers. Dai et al. also found that postoperative adjuvant chemoradiotherapy is effective for stage III GC, regardless of the MSI status (34). The conclusion of our study, if validated, provides support for peri-operative chemotherapy in cStage III patients with MSI-H GCs. We can speculate that different therapeutic strategies should be adopted for stage II and III MSI-H GCs.

Immunotherapy (anti-programmed cell death-1 inhibitor) has been considered a promising option for MSI-H GCs (35). Although immunotherapy has been found to be effective for MSI-H refractory or metastatic tumors (36), evidence is lacking to use it to replace chemotherapy for cStage III MSI-H GC. Zheng et al. reported a high pathological CR rate (83.3%) of MSI-H gastrointestinal tumors treated by neoadjuvant immunotherapy, but this was a case series with only six cases (37). Thus, there is an urgent need to find an efficient multimodality treatment (may be taxane-based chemotherapy regimens ± immunotherapy) for cStage III MSI-H GC.

Our study has several limitations. Firstly, this was a retrospective single-center study, including Chinese patients only. Since a large proportion of Chinese patients have an advanced tumor stage (usually cStage III) at diagnosis, it is important to explore better solutions for this entity of patients in China. Secondly, we did not test MSI status on pre-therapeutic biopsy tissue. Of note, Kohlruss et al. found that MSI status was consistent with resected tumors in all 42 biopsy samples (10), in line with our speculation that NAC would not change microsatellite status. Thirdly, regimens of NAC were heterogeneous due to the retrospective aspect of our study. Well-designed prospective studies are needed to validate our findings. Fourthly, the median follow-up was short; the median DFS has been reached in the study cohort (21.6 months) but not the median overall survival (OS). So we chose DFS instead of OS to compare between MSI-H and MSS groups. Longer follow-up is necessary to establish any convincing conclusion about survival. Fifthly, as the survival curves of Grades 2 and 3 were similar in our study, we merged TRG 2 and 3 tumors into the same poor-responders category [in accordance with (21)], but this may have skewed our results.

In conclusion, MSI-H was associated with poorer histological regression after NAC in clinical stage III GC. However, better survival was found in these patients compared to MSS/MSI-L patients. TRG evaluation had prognostic significance in MSS/MSI-L patients but not in MSI-H patients. We suggest that MSI status testing be used to predict survival for cStage III patients treated by NAC and curative gastrectomy. However, further studies are needed to assess the value of NAC for cStage III GC patients with MSI-H phenotype.

## Data Availability Statement

The raw data supporting the conclusions of this article will be made available by the authors, without undue reservation.

## Ethics Statement

The studies involving human participants were reviewed and approved by the Ruijin hospital ethics committee. Written informed consent for participation was not required for this study in accordance with the national legislation and the institutional requirements.

## Author Contributions

ZC, SL, ZZ, and MZ contributed to the conceptualization. SL, AF, JS, and LZ contributed to the methodology. ZC, WR, and JM contributed to the formal analysis and investigation. JS, JM, and LZ contributed to the data curation. MZ, JS, and LZ contributed to the funding acquisition. ZC, SL, and WR contributed to writing of the original draft. MZ, AF, and ZZ contributed to reviewing and editing. ZC, WR, and SL should be considered joint first authors. MZ, ZZ, and LZ should be considered joint corresponding authors. All authors contributed to the article and approved the submitted version.

## Funding

This work was funded by the Shanghai Municipal Key Clinical Specialty (grant number shslczdzk00102 to MZ), Shanghai Municipal Health Commission (grant number 2019SY030 to LZ), and Shanghai Science and Technology Committee (grant number 18411953200 to JS).

## Conflict of Interest

The authors declare that the research was conducted in the absence of any commercial or financial relationships that could be construed as a potential conflict of interest.

## Abbreviations

GC, gastric cancer; LN, lymph node; NAC, neoadjuvant chemotherapy; MSI-H, microsatellite instability-high; CSCO, Chinese Society of Clinical Oncology; AEG, adenocarcinoma of esophago-gastric junction; TAP-CT, thoracic-abdominal-pelvic computed tomography; UICC, Union for International Cancer Control; AJCC, American Joint Committee on Cancer; RECIST, Response Evaluation Criteria in Solid Tumors; CR, complete response; PR, partial response; SD, stable disease; PD, progressive disease; ORR, objective response rate; DG, distal gastrectomy; TG, total gastrectomy; THG, transhiatal extended gastrectomy; TRG, tumor regression grade; MSI-L, microsatellite instability-low; MSS, microsatellite stability; dMMR, mismatch repair deficiency; ECOG, Eastern Cooperative Oncology Group; DFS, disease-free survival; pMMR, mismatch repair proficiency; OS, overall survival.
